# Application of Piezoelectric Material and Devices in Bone Regeneration

**DOI:** 10.3390/nano12244386

**Published:** 2022-12-09

**Authors:** Chunyu Yang, Jianying Ji, Yujia Lv, Zhou Li, Dan Luo

**Affiliations:** 1 Beijing Institute of Nanoenergy and Nanosystems, Chinese Academy of Sciences, Beijing 100083, China; 2 State Key Laboratory of Heavy Oil Processing, College of New Energy and Materials, China University of Petroleum (Beijing), Beijing 102249, China; 3 Center on Nanoenergy Research, School of Physical Science and Technology, Guangxi University, Nanning 530004, China

**Keywords:** piezoelectric materials, electrical stimulation, devices, biomedical engineering applications

## Abstract

Bone injuries are common in clinical practice. Given the clear disadvantages of autologous bone grafting, more efficient and safer bone grafts need to be developed. Bone is a multidirectional and anisotropic piezoelectric material that exhibits an electrical microenvironment; therefore, electrical signals play a very important role in the process of bone repair, which can effectively promote osteoblast differentiation, migration, and bone regeneration. Piezoelectric materials can generate electricity under mechanical stress without requiring an external power supply; therefore, using it as a bone implant capable of harnessing the body’s kinetic energy to generate the electrical signals needed for bone growth is very promising for bone regeneration. At the same time, devices composed of piezoelectric material using electromechanical conversion technology can effectively monitor the structural health of bone, which facilitates the adjustment of the treatment plan at any time. In this paper, the mechanism and classification of piezoelectric materials and their applications in the cell, tissue, sensing, and repair indicator monitoring aspects in the process of bone regeneration are systematically reviewed.

## 1. Introduction

Due to diseases, car accidents, etc., bone damage has become a common condition. Bone is a high-density connective tissue composed of cells, an extracellular matrix (ECM), and bone colloidal fibers. Bone is divided into dense bone and cancellous bone. The key feature of bone that distinguishes it from other tissues is that it has a large amount of calcium salt deposits in its extracellular matrix, which gives it a hard characteristic [1,2]. Osteocytes are divided into three types of cells, namely, osteoblasts, osteoclasts, and osteocytes. Osteoblasts can secrete three times the volume of matrix in three to four days, and then become osteocytes ambushed in them. Bone cells are the main components of bone tissue. Osteoclasts, together with macrophages, can absorb bone. Due to the presence of different species of cells in the ECM, the ECM of bone is stiff, whereas the ECM of cartilage is soft. Meanwhile, all connective tissues, including bone, are highly vascularized [3]. When performing bone restoration, the gold standard is autologous bone grafting. However, autologous bone grafting has the problem of insufficient bone sources and can easily lead to secondary injury. Contemporary treatment methods include fixing the injury site to allow it to grow on its own; researchers have also developed bone grafting combined with appropriate external stimulation such as electrical stimulation, ultrasound stimulation, a gradient hypoxic environment, etc.

Electricity is very important for living organisms. Muscle contraction and nerve impulse induction all require the presence of electrical signals. Since the last century, researchers have discovered that there is a piezoelectric effect in bone, which generates an electrical signal when stress is applied to the bone. The electrical signals in bone come from collagen [4]. When electrical signals stimulate the bone, this stimulates cell surface membrane proteins or stimulates the Ca^2+^ voltage-gated channel on the cell membrane surface, resulting in a change in the intracellular and extracellular concentration of Ca^2+^. Direct current stimulation can also promote the cell secretion of prostaglandins (PGE2), morphogens, and growth factors, thereby affecting the cell [5]. Therefore, electrical stimulation has a positive effect on bone tissue [6].

Currently, the most commonly used clinical means of treating bone injury are long-term fixation, bone grafting, and intramedullary nailing. Electrical stimulation was introduced in fracture treatment in the 1980s, and studies have shown that direct current stimulation of approximately 1 Hz has a good effect on fracture treatment. Traditional invasive methods of electrical stimulation require at least two surgeries—one to implant the electrode and one to remove the electrode—easily resulting in bone infection. Percutaneous leads and external power can also cause inconvenience during treatment.

The use of piezoelectric materials can effectively simulate the internal electrical environment of bone and effectively promote the healing of bone damage. Additionally, piezoelectric materials can produce electrical stimulation without an external power supply, exhibiting characteristics of convenience and good biological adaptability. Piezoelectric materials were first used as orthopedic implants in the 1980s. Piezoelectric materials used as scaffold materials include piezoelectric ceramics, piezoelectric polymers, or piezoelectric ceramic–polymer composite materials. Moreover, some piezoelectric devices can detect the health of bones through mechanical electrical impedance technology, including whether the bones are in a state of osteoporosis or whether bone injuries have healed, and can also promote bone healing by providing low-magnitude, high-frequency (LMHF) vibration [7,8]. As a kind of smart material [9], piezoelectric material can realize the mutual conversion of electrical and mechanical signals and can be used as a brake and a sensor at the same time; while using it as a brake, it can generate electrical signals by using the kinetic energy generated by the movement in the body. It is environmentally friendly because it does not require an external power supply, and it is economically friendly because it is possible to avoid secondary removal surgery by using a biodegradable piezoelectric material. Additionally, the material itself is designable, and there is plenty of room for cost reduction.

This review focuses on the application of the piezoelectric effect in bone regeneration, starting with piezoelectric materials and piezoelectric devices. The latest advances in recent years at the cellular level and tissue level, as well as in detecting and monitoring the effectiveness of treatment, are reviewed. This review will inspire subsequent bioengineering researchers to develop more advanced materials and technologies and promote clinical translation.

**Figure 1:**
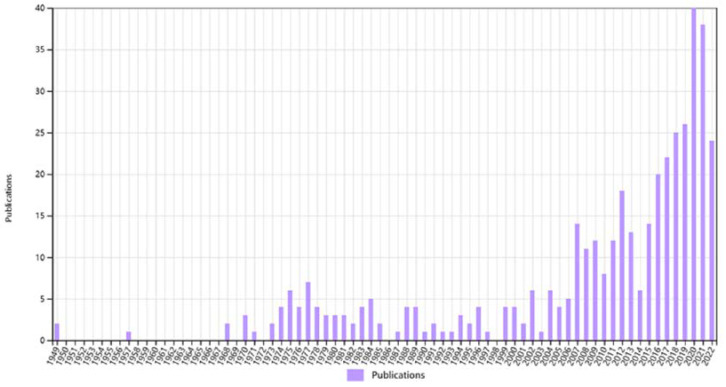
A bibliometric study based on Scopus or Web of Science database.

Using “bone” and “piezoelectricity” as title words to search on the Web of Science shows the below results (Figure 1). As you can see from this graph, there has been a particularly large amount of research combining piezoelectricity with bone in the last decade, which reveals that this is a very promising area of research.

## 2. Classification and Mechanism of Piezoelectric Material and Devices

### 2.1. Mechanism of the Piezoelectric Effect

**Figure 2:**
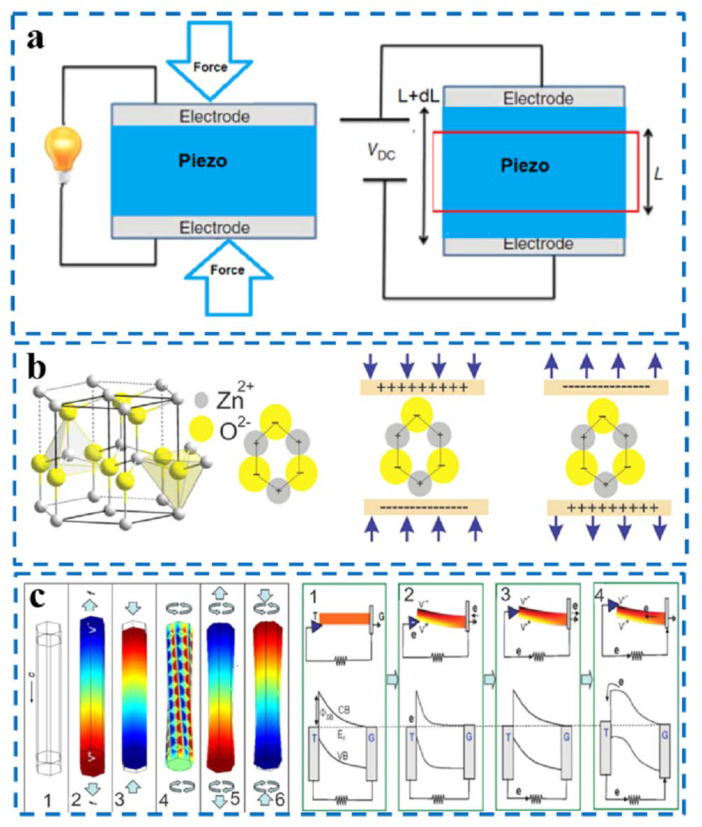
(**a**) Direct and reverse piezoelectric effects; (**b**) ZnO wurtzite structural model, with the piezoelectric potential in compression and tensile mode; (**c**) piezo-potential distribution in ZnO nanowires (left 1–6) and band diagrams in nanogenerators (right 1–4) [13].

The phenomenon of piezoelectricity was first discovered by the Curie brothers in 1880 by identifying the phenomenon that electric charge is generated when force is applied to quartz crystals and Rochelle salt [10]. In the 1950s, scientists discovered that there is also a piezoelectric effect in biological tissues [11], and in the 1980s, scientists first used piezoelectric materials as an option for bone implant materials [12]. The piezoelectric effect is divided into the direct piezoelectric effect and reverse piezoelectric effect (Figure 2a) [13]: the direct piezoelectric effect refers to the mechanical force applied to the material leading to the polarization of the dipole moment inside the material to generate electric charges, whereas the reverse piezoelectric effect refers to the mechanical deformation of the material itself under the action of the electric field.

Polarization is generated by corona polarization and thermal polarization: thermal polarization can be polarized in oil/liquid and can also be polarized in the air, which is generally used for the polarization of biological materials to prevent pollution. In addition, the value of voltage and temperature depends on the material itself and the degree of target polarization. The d_ij_ (piezoelectric constant) is used to characterize the piezoelectric properties of piezoelectric materials: its value refers to the amount of charge generated when stress is applied to the material, or the mechanical stress generated under a unit electric field. The subscript ‘i’ refers to the direction of polarization of the dipole moment, or the direction of the applied electric field; the subscript ‘j’ refers to the direction in which strain is generated or the stress is applied [6].

The most classic piezoelectric material is zinc oxide (ZnO). The wurtzite structure of ZnO is shown in Figure 2b [13], exhibiting many tetrahedrons with Zn^2+^ and O^2−^ vertices stacked in parallel and alternately. When no external force is present, its positive and negative charge centers are coincident. However, in the presence of an external force, its positive and negative charge centers become dipoles that can generate piezoelectric potential. The potential distribution of ZnO nanowires (NWs) under the action of external force is shown in Figure 2c [13]. Here, 2, 3, and 4 show the potential distribution under tensile, compressive, and rotational forces, respectively; 5 and 6 indicate the joint action of tensile force and rotational force and the combination of compression force and rotational force, respectively. The relationship between the piezoelectric potential and the output current of a piezoelectric nanogenerator is shown in Figure 2c [13], when the atomic force microscopy tip (T) pushes from the low end of the surface of ZnO nanowires (NWs) to the top, causing electrons to flow in the loop, resulting in an output of current.

### 2.2. Classification of Piezoelectric Material

Piezoelectric materials are crystalline materials that can convert mechanical to electrical energy when subjected to pressure. More and more piezoelectric materials are being developed and manufactured to meet the needs of different applications. These materials can be classified into four main categories according to their chemical composition and physical structure: piezoelectric single crystals [14], piezoelectric ceramics [15], piezoelectric polymers [16], and composite piezoelectric materials [17].

#### 2.2.1. Piezoelectric Single Crystals

**Figure 3:**
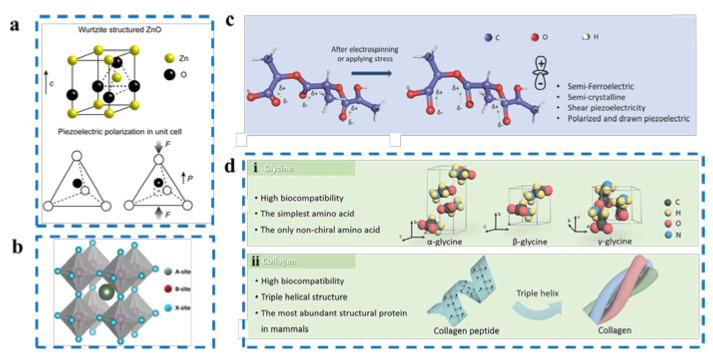
(**a**) Schematic diagram of ZnO piezoelectric mechanism [14]; (**b**) schematic diagram of the structure of chalcogenide [18]; (**c**) schematic diagram of the piezoelectric mechanism of piezoelectric polymers [16]; (**d**) schematic diagram of the piezoelectric mechanism of bio-piezoelectric materials (i) glycine; (ii) collagen [19].

Piezoelectric single crystals—crystals developed in a long-range, organized way in accordance with the dot matrix in crystal space—are generally referred to as piezoelectric crystals [18,19,20]. Their crystal structure lacks a center of symmetry, and when subjected to mechanical stress from the outside, the positive and negative charge centers within the crystal are moved and polarized, causing the accumulation of charges on some of its surfaces that are linearly proportional to the force, of equal magnitude, and the opposite sign, resulting in piezoelectricity (Figure 3a) [14]. Piezoelectric crystals include GaN (gallium nitride) [21,22], SiC (silicon carbide) [23], ZnO [24,25], etc.

#### 2.2.2. Piezoelectric Ceramics

Piezoelectric ceramics are polycrystalline piezoelectric materials that are generally ferroelectric. The spontaneous polarization direction of each grain in the ceramic is haphazard, and the piezoelectric effects between the grains cancel each other out. Piezoelectric ceramics without polarization treatment do not show piezoelectric effects macroscopically. Therefore, to synthesize piezoelectric ceramics with a piezoelectric effect, a strong DC (direct current) electric field must be added to the ceramics so that the electric domains of each grain are turned along the electric field direction. Compared with piezoelectric monocrystals, piezoelectric ceramics have the advantages of a simple preparation process, strong piezoelectricity, high dielectric constant, can be made into arbitrary shape components, have a low cost, and are suitable for mass production. In recent decades, piezoelectric ceramics have been widely used in electronic information, integrated circuits, computers, aerospace, marine mapping, automotive, and energy, as well as other parts of daily life [26,27]. For instance, steady piezoelectric and dielectric qualities are often necessary for piezoelectric ceramics to function as piezoelectric oscillators. Piezoelectric ceramics are required to have a high electromechanical coupling coefficient and a large dielectric constant to achieve the conversion of mechanical and electrical energy in a transducer application. Using conventional inorganic chalcogenide materials such as barium titanate (BTO and BaTiO_3_) [18] and lead zirconate titanate (PZT and PbZrxTi1-xO_3_) [15] created for various applications such as capacitors, piezoelectric devices, and ferroelectric devices, thousands of compounds with a chalcogenide structure (ABX_3_, where A and B are cations and X is an anion, respectively) have been reported (Figure 3b) [28].

#### 2.2.3. Piezoelectric Polymers

The piezoelectricity of piezoelectric polymers is mainly due to the ferroelectric phase with spontaneous polarization or the crystalline phase with asymmetric centers. Through mechanical stretching or high-temperature and high-voltage DC polarization, the disordered dipole orientation within the piezoelectric polymer crystals can be arranged to achieve excellent piezoelectricity [16]. Under the action of external mechanical forces, strain is induced within the piezoelectric polymer to order the dipoles in the direction of the force, thus inducing an electric charge on both surfaces of the material and converting the weak mechanical vibration energy into electrical energy (Figure 3c) [16,29]. The piezoelectric polymer materials that have been studied and widely used include polyvinylidene fluoride (PVDF) [30,31] and its copolymer (PVDF-TrFE) [32], terpolymer (PVDF-TrFE-CFE) [33], and poly(levulinic acid) (PLLA) [34], with good biocompatibility. Piezoelectric polymer materials have the advantages of good flexibility, excellent processability, low density, low impedance, high piezoelectric coefficient, and good biocompatibility, and they are widely used in the fields of pressure sensing, energy harvesting, and biomedicine [35,36,37].

#### 2.2.4. Bio-Piezoelectric Materials

In 1941, Martin discovered that, when wool and hair rubbed against each other, the induced positive and negative frictional charges changed with the direction of friction [38]. This effect is caused by fiber epidermal cells and was the first time that a biological material was found to have piezoelectric properties. In 1957, Fukada studied the piezoelectricity in dry bones, showing that it originated from the in-plane piezoelectric effect of collagen; this experimental result greatly contributed to the progress of the study of bio-piezoelectricity. The study of the piezoelectric effect in bone has been the focus of bio-piezoelectricity research since then [39]. Subsequently, Fukada et al. investigated the piezoelectricity of the Achilles tendon and concluded that its piezoelectricity mainly comes from collagen fibers arranged along the long axis of the Achilles tendon and solved the piezoelectric coefficient matrix of collagen fibers [40]. In addition to the hard tissues in living organisms, researchers have conducted numerous studies on soft tissues in living organisms, including blood vessels [41], skin [42], muscles [43], and nerves [44]; the results have shown that a large number of soft tissues have piezoelectric properties. The piezoelectricity of biological tissues is generally considered to originate from oriented biopolymer molecules. Oriented long-chain fiber molecules undergo the deformation of crosslinks in the molecule after being subjected to shear stress, causing charge displacement (Figure 3(dii)). Fukada et al. further showed, through studies on synthetic peptides and optically active polymers, that the internal rotation of a large number of dipoles formed by -CO and -NH leads to piezoelectric effects in biomaterials [45]. The structure of their side chains determines how distinct amino acids differ from one another. Glycine, for instance, crystallizes into three distinct structures, depending on the crystallization circumstances (Figure 3(di)). Glycine crystals have crystal symmetry and are thus not piezoelectric. Glycine has ferroelectric properties and non-centrosymmetric crystal structures [19]. Biopiezoelectric materials have a wider range of uses in biomedicine because they are more biocompatible than conventional piezoelectric materials.

#### 2.2.5. Composite Piezoelectric Materials

Piezoelectric composites usually refer to piezoelectric materials obtained by dispersing inorganic piezoelectric materials with nanostructures (e.g., nanoparticles, nanowires, nanosheets, etc.) into a matrix of piezoelectric polymers by a simple preparation process [46]. Common piezoelectric composites include lead zirconate titanate (PZT)/polymer [47,48], lead titanate (PT)/polymer, etc. This composite material combines both the excellent piezoelectricity of organic piezoelectric materials and the flexibility and fatigue resistance of piezoelectric polymers, improving the shortcomings of single piezoelectric materials. In addition, there is great flexibility in material compound selection, and these advantages expand the scope of the application of piezoelectric composites in the field of flexible devices [17]. For example, the PVDF/SiC composites prepared by Rasoolzadeh et al. have greatly enhanced piezoelectric properties due to higher β-phase fraction and improved charge transfer near the semiconductor SiC nanoparticles [49].

### 2.3. Classification of Piezoelectric Devices

Piezoelectric devices usually refer to a class of electronic or optoelectronic devices based on the piezoelectric effect, and their most basic regulatory units are usually called piezoelectric transistors. They can roughly be divided into two categories according to their structure and operating mechanism, namely, field-effect transistors and piezoelectronic transistors.

**Figure 4:**
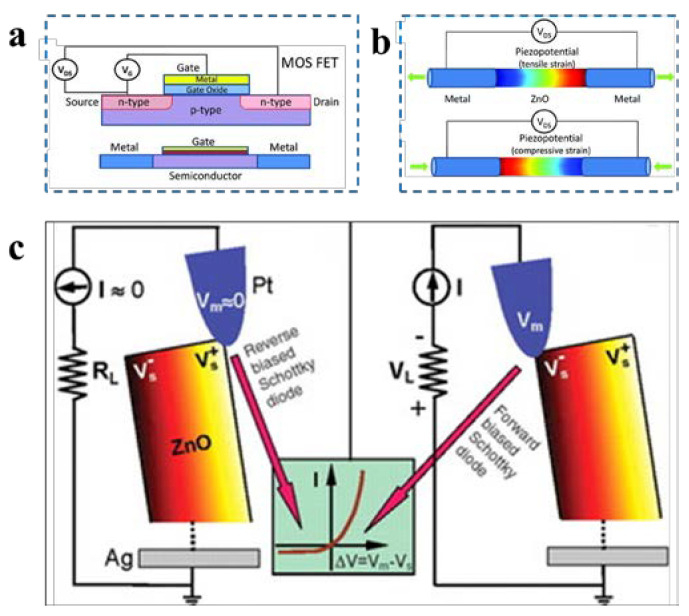
(**a**) Schematic diagram of a semiconductor nanowire field-effect tube. (**b**) Schematic diagram of a piezoelectric transistor [50]. (**c**) Working principle of a ZnO nanogenerator [65].

Conventional field effect transistors usually adopt the basic three-electrode structure of the source, drain, and gate. The principle is to apply a driving voltage signal between the source and drain, and then provide a gate voltage signal to regulate the channel width of carriers in the field effect transistor to achieve the regulation of the electrical transport characteristics of the transistor (Figure 4a) [50]. With the extensive and intensive research in the field of piezoelectrics, many piezoelectric semiconductor materials have been used to fabricate piezoelectric transistors, such as ZnO (nanowires [51], thin films [52]), GaN (nanorods [53], nanowires [54], nanoribbons [55], nanotubes [56]), CdS (nanowires [57]), CdSe (nanowires [58,59]), InAs (nanowires [60]), InN (nanopillars [61]), and other crystal structures such as ZnSnO_3_ (nanowires [62,63]) and CdTe (nanowires [64]). In addition, transition metal sulfides (TMDCs), which have a centrosymmetric crystal structure in the bulk state, have been found to exhibit piezoelectric effects at the atomic thickness layer, thus providing a new low-scale material option for piezoelectronic devices.

Unlike conventional field-effect transistors, piezoelectric transistors use the piezoelectric potential generated by piezoelectrically polarized bound charges as the gate voltage signal to regulate the carrier transport characteristics of the transistor instead of the gate voltage (Figure 4b) [50]. A typical representative is the piezoelectronic nanogenerator. In 2006, piezoelectric nanogenerators were first proposed [65], using the tip of an atomic force microscope to poke a ZnO nanowire and measuring a nanowire piezoelectric output signal of 8 mV. The output power of a single ZnO nanowire is very small. To increase the output power of the piezoelectric nanogenerator, as well as to move away from the dependence on AFM and transform the piezoelectric nanogenerator from a concept to a practical technology, an innovative design of the structure of the piezoelectric nanogenerator is required. Based on the existing piezoelectric nanogenerator designs, piezoelectric nanogenerators can be broadly classified into two categories: Schottky-contact-structure-based piezoelectric nanogenerators, and sandwich-structure-based piezoelectric nanogenerators. Taking ZnO as an example, the operating principle of ZnO nanogenerators exploits the coupling of the semiconductor properties of ZnO material with piezoelectric properties. The initial study of the piezoelectric properties of ZnO utilized ZnO nanorods (Figure 4c) that were allowed to bend under the action of an AFM probe. This deformation causes a certain deviation of Zn^2+^ and O^2-^, whose original centers of symmetry coincide within ZnO, to generate a piezoelectric potential, with the stretched side of the nanorod gathering a positive charge, and thus a positive potential, and the compressed side gathering a negative charge, i.e., a negative potential [65].

## 3. Bone Regeneration Based on Piezoelectric Material and Devices

### 3.1. Piezoelectric Materials and Devices Applied in Cells

Bone healing is the process of repairing a fracture or bone defect, which is essentially a regenerative process after a bone injury. Ideally, after the repair process, only the bone tissue is rebuilt without scar formation. However, studies have found that 5% to 10% of patients still suffer damage during the bone healing process, resulting in delayed bone healing or the non-healing of bone. Cell proliferation and differentiation are essential biological processes in the bone healing process. The cells of bone tissue mainly include bone marrow mesenchymal stem cells (BMSCs), osteoblasts, osteocytes, and osteoclasts. Only osteocytes are present within the bone tissue, while the other three types of cells are located at the edges of the bone tissue. Currently available studies on the piezoelectric stimulation of bone tissue cells have focused on osteogenic and osteoblastic cells. The state of the cells can be changed by the stimulation of tissue cells, which can modify the Na^+^ (Ca^2+^) permeability of excitable cells, resulting in action potentials and changes in the internal environment brought on by changes in the Na^+^ and K^+^ ion channels. Endogenous electrical signals play a critical role in controlling cell fate, tissue development, and regeneration. The endogenous direct current electric field (DC EF), which is typically produced by intracellular ion transport, plays an indispensable role in maintaining and manipulating the normal physiological function and activity of the ECM. Reactive bioelectrical impulses, in particular, affect the behavioral guidance of cells in bone damage.

**Figure 5:**
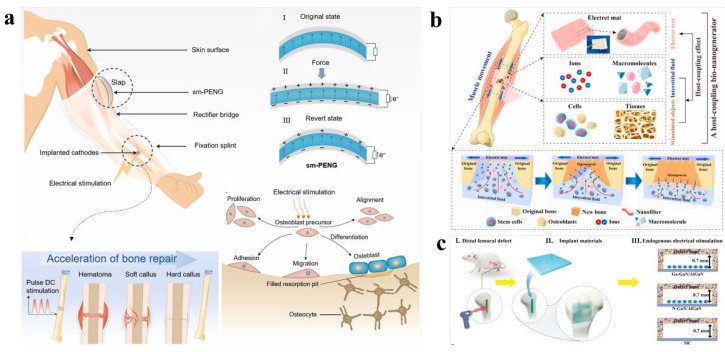
(**a**) Schematic diagram of self-powered electrical stimulation for bone repair [66]. (**b**) Schematic diagram of an electret-based HCBG implanted onto a bone injury in vivo [67]. (**c**) In vivo bone defect repairing ability of GaN/AlGaN films [68].

Osteoblasts, in particular, release a variety of bioactive compounds that regulate and impact the process of bone formation and reconstruction. Osteoblasts are primarily differentiated from mesenchymal progenitor cells in the stroma of the inner and outer periosteum and bone marrow. The differentiation and value-added of osteoblasts can be successfully aided by electrical stimulation. In the field of orthopedics, promoting bone formation using currents of 5 to 100 microamps has been found to have beneficial effects. Osteoblasts can specifically secrete a variety of bioactive substances that regulate and influence the process of bone formation and reconstruction. In order to play a significant role in an electrical stimulation treatment system for osteogenic differentiation, Zhang et al. created a self-powered pulsed DC stimulation device for bone repair that integrates a thermally processed, shape memory compression-based, arch-shaped structured electrical nanogenerator (sm-PENG) and a fracture fixation splint (Figure 5a) [66]. Under long-term culture conditions, the sm-PENG can effectively promote cell proliferation and alkaline phosphatase (ALP) activity of the cells which, in turn, promotes calcium deposition, the extracellular mechanism of mineralization, and osteogenic differentiation. The short-circuit current of sm-PENG is up to 20 μA. This device has a wide range of potential applications in bone restoration.

BMSCs can differentiate into tissue cells of mesodermal and neuroectodermal origin, including myogenic cells, hepatocytes, osteoblasts, chondrocytes, fibroblasts, glial cells, neuronal cells, hematopoietic stem cells, and stromal cells. Bone marrow mesenchymal stem cells alone can better promote fracture healing and cartilage injury repair. Yu et al. designed an implantable self-powered generator (ISPG) to address the problem of energy supply for driving electronic devices and electrical stimulation therapies in vivo (Figure 5b) [67]. This ISPG is a self-powered regional electrical-environment-configured host-coupled bio-nanogenerator (HCBG) for bone regeneration. The implanted matrix fluid and the stimulated object are connected to the HCBG, which features a porous electret nanofiber mat, producing a coupling effect. This bio-nanogenerator not only eliminates the drawbacks of conventional ISPG but also accomplishes electrical stimulation therapy and biomechanical energy scavenging. The ability of bone marrow mesenchymal stem cells to differentiate into osteoblasts in vitro and regenerate bone in vivo was greatly improved. Li et al. induced endogenous electrical stimulation using polarity-controlled GaN/AlGaN materials to improve bone regeneration (Figure 5c) [68]. It was possible to create charged GaN/AlGaN surfaces with opposing polarity and zeta potentials within the physiological potential range by manipulating the direction and amplitude of piezoelectricity and spontaneous polarization in the functional layer (GaN). In vivo, Ga-polar GaN/AlGaN nanofilms (negatively charged surfaces) demonstrated faster and more effective bone healing than N-polar GaN/AlGaN (positively charged surfaces). Additionally, the in vitro adhesion, migration, recruitment, and osteogenic differentiation of bone marrow mesenchymal stem cells were all greatly aided by Ga-polar GaN/AlGaN heterostructures. Piezoelectric biomaterials are being developed and used to cure bone abnormalities more frequently. Recent advances have made electret materials into promising electroactive materials. The polarized charge of electret material is held inside or on the surface after being polarized with an external electric field, generating a steady built-in potential that produces endogenous electrical stimulation. For bone regeneration, Qiao et al. created a sandwich-shaped SiO_2_ electret coupled with a poly(dimethyl siloxane) (SiO_2_/PDMS) electroactive membrane [69]. By providing steady and long-lasting endogenous electrical stimulation and reducing potential degradation compared with pure PDMS membranes, electret SiO_2_/PDMS membranes can effectively stimulate bone regeneration. This electret composite membrane exhibits a steady biopotential in surface potential measurements, which can effectively boost cellular activity and accelerate the osteogenic differentiation of BMSCs. This membrane has excellent therapeutic applicability in orthopedic and craniofacial surgery. It is well known that controlling the spontaneous and piezoelectric polarization of GaN/AlGaN enables the effective control of its surface polarity (SP and PE, respectively).

### 3.2. Piezoelectric Material and Devices Applied in Tissue

Bone tissue is composed of a variety of bone cells including the extracellular matrix. Previously, piezoelectric materials have focused on promoting cell proliferation, differentiation, and migration. However, in practical applications, the shape, size, type, and characteristics of the bone tissue need to be taken into account to design materials that are targeted to achieve better bone regeneration results. Here, we summarize several typical piezoelectric materials designed for bone regeneration applications based on tissue characteristics.

**Figure 6:**
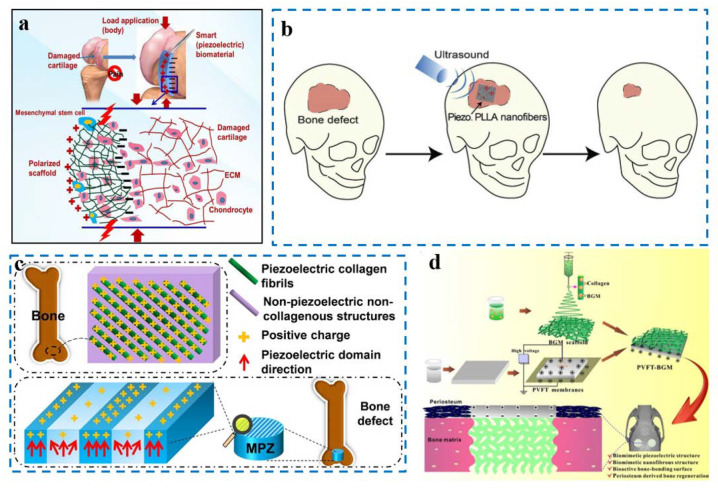
(**a**) Manufacturing processes and bionic applications of degradable piezoelectric scaffolds and the induction of chondrocytes based on the relationship between degradation and piezoelectric effects [70]; (**b**) biodegradable piezoelectric PLLA nanofibers are used in combination with non-invasive ultrasound (US) to generate stable electrical stimulation for bone regeneration [71]; (**c**) MPZs were constructed to mimic piezoelectric microregions in native bone for bone defect repair (MPZs were manufactured by setting selective laser irradiation and polarization on the KNN surface, then implanted to a bone defect model to assess bone regeneration) [72]; (**d**) schematic diagram of the manufacturing process and potential advantages of PVFT-BGM scaffold for bone regeneration [73].

Cartilage is tissue located at the end of the bone that forms a joint and is a soft and flexible spongy structure. Once the cartilage is injured, it is difficult to recover because it does not exhibit factual vascularization and has a complex structure with viscoelasticity and anisotropy. In clinical practice, cartilage injury often leads to chronic pain, and simple medication does not help. However, treatments such as autologous or allogeneic osteochondral grafts have limitations in the size of injured cartilage and donor zone infection; the most common contemporary treatment is still joint replacement surgery. Chondrocytes and mesenchymal stem cell (MSC)-based tissue engineering scaffolds are widely reported in the literature, and the application of external physical stimuli by smart biomaterial scaffolds can be translated into various signals which can be recognized by the cells. Piezoelectric materials can generate electrical signals when mechanical loads are applied, which ultimately stimulate growth factor synthesis on the cell surface through calmodulin. The PHBV copolymer has low cytotoxicity, good piezoelectricity, and a longer degradation time, making it suitable for use in tissue engineering for cartilage regeneration. Barium titanate is another common piezoelectric material with high piezoelectric properties. Hence, Jacob et al. used an electrostatic spinning technique to simulate the structure and piezoelectric coefficient of natural cartilage using poly-(3-hydroxybutyrate-co-3-hydroxyvalerate) (PHBV) doped with BaTiO_3_ (Figure 6a) [70]. The ability to not only promote the proliferation, migration, and growth of human mesenchymal stem cell-derived chondrocytes, but also the expression of the collagen II gene, has been demonstrated experimentally. Compared with unpolarized pure PHBV material or without BaTiO_3_, this piezoelectric scaffold can effectively promote the regeneration of cartilage.

The surface of the biological scaffold is charged; thus, it can adsorb beneficial proteins [74], thereby stimulating a variety of protein pathways and achieving the purpose of stimulating osteogenesis [75]. In addition, the surface of bone tissue is inherently charged; therefore, linking electrical signals to biological scaffolds can facilitate the effect of osteogenesis [76]. Due to the characteristics of piezoelectricity, piezoelectric materials can generate electrical signals through the piezoelectric effect under mechanical movement [77], avoiding the drawbacks of percutaneous wires and applied batteries, resulting in a higher application value in osteogenesis. Ritopa Das et al. designed a biological scaffold that combines piezoelectric nanofiber PLLA (Poly(L-lactic acid)) and vitro ultrasound stimulation to achieve the effect of remote-controlled electrical stimulation without the need for batteries (Figure 6b) [71]. In addition, PLLA material is biodegradable, and the degradation time is relatively long, which can match the time of osteogenesis. Although there are PLLA scaffolds for bone regeneration in cats, these are applied through the passive movement of cats. This material can control the time and quantity of electrical signals generated by ultrasound. Using electrospinning to manufacture piezoelectric nanofibers, materials with different piezoelectric signals were produced by controlling the speed of a collector drum, and the output of the piezoelectric signal for 26 days was evaluated in vitro, indicating the long-term effectiveness of the material. In vitro experiments to promote the osteogenic differentiation of stem cells—by measuring the alkaline phosphatase (ALP), the Alizarin red assay, and the expression of osteocalcin and osterix osteogenic genes—proved that the stronger the piezoelectric signal (the material which uses higher speed in the manufacturing process and the using of ultrasound), the better the osteogenic differentiation. Subsequently, a mouse skull defect model experiment was carried out, in which a 3.5 mm bone defect was made in the mouse skull, then the material was placed and ultrasound treatment was performed. Finally, X-ray imaging and nuclear fast red ALP staining, as well as the expression of Collagen 3.6-GFP-topaz fluorescent reporter genes and toluidine blue staining showed that the effect of piezoelectric materials plus ultrasound is optimal for the repair of bone defects.

The microenvironment in which the bone is located can be regarded as an area composed of micro-regions of piezoelectric collagen materials and non-piezoelectric non-collagen materials [78]. To imitate the electrical signal of this scale, Peng Yu et al. used K_0.5_Na_0.5_NbO_3_ (KNN) ceramics, and by selectively performing laser irradiation, the mixed phase of the quadrature-phase and the tetragonal phase with high voltage became a tetragonal phase with low-voltage electricity (Figure 6c) [72]. Thus, the material surface of the high-voltage electrical region and the low-voltage electrical region that simulates the bone microenvironment was formed. Compared with unaltered KNN ceramics, it was demonstrated that the markers of osteogenic differentiation, Runt-related transcription factor 2 (Runx2), and ALP were more expressed in cells cultured on the surface of KNN ceramics with high- and low-voltage regions, indicating that the surface of the material similar to bone piezoelectric microstrips was more conducive to osteogenic differentiation. Then, conducting a rabbit femoral condyle implantation experiment, the micro-CT results showed that the microscale piezoelectric structures (MPZs) had the best osteogenic performance compared with the control group of KNN and hydroxyapatite (HA).

The difficulty of treatment varies with the size of the bone defect. In small bone defects, the defect site is rapidly covered by periosteum, which encourages osteoblasts to migrate, proliferate, and differentiate at the opening wound. This results in the rapid formation of new bone at the defect site. Bone defects exceeding a critical size are not completely covered by the periosteum, leading to slow bone healing or osteointegration. In response, Zhao et al. designed a novel bio-scaffold material to mimic the periosteal structure and bone microenvironment. By loading bioactive glass micro-nano particles onto poly(vinylidene fluoride-trifluoroethylene), a combination of piezoelectric polymers and bioactive glass nanofibers (PVFT-BGM) was made to simulate the periosteal structure (Figure 6d) [73]. The piezoelectric polymer is responsible for providing electrical signals to simulate the electrical microenvironment of bone. Bioactive glass micro-nano particles with Ca^2+^, phosphorus ions (P^4+^), and other mineral ions on them can be released, which facilitates the formation of bone minerals. Further mechanistic studies have confirmed that PVFT-BGM activates Ca^2+^-sensitive receptors (CaSR) in osteoblasts while affecting downstream signaling pathways. The ultimate goal of promoting the growth, proliferation, and differentiation of bone marrow stem cells, and the formation of periosteal-like tissue and bone regeneration, was observed in animal experiments.

### 3.3. Piezoelectric Material and Devices Applied in Sensing and Repair Indicator Monitoring

**Figure 7:**
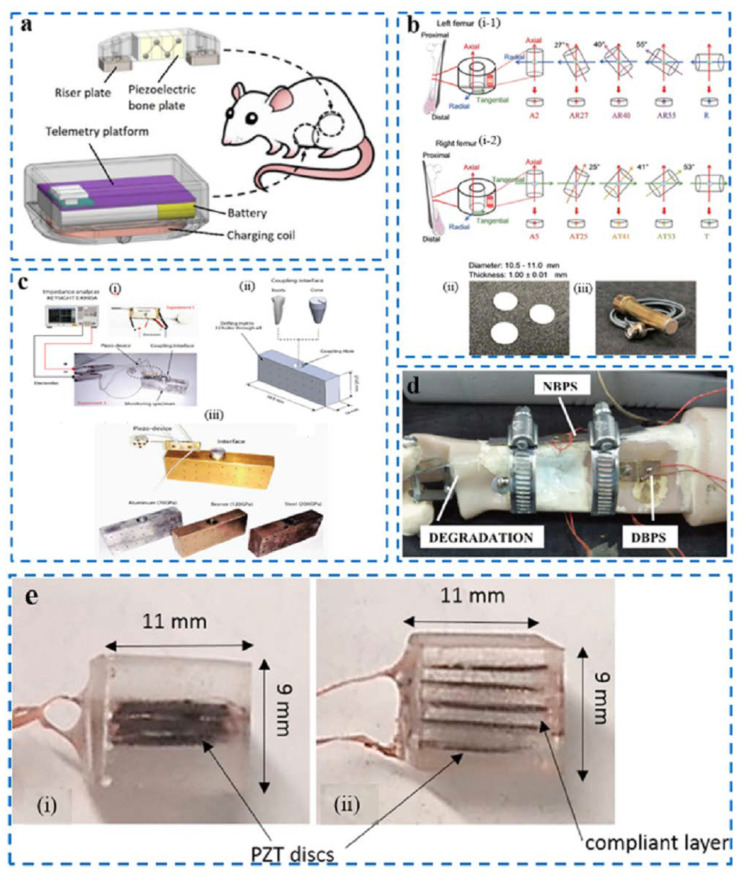
(**a**) Piezoelectric fixation plate mounted on the femur of a rat [81]; (**b**) (i) preparation of bone samples, (i-1) bone plate sample of left femoral specimen, (i-2) bone plate sample of right femoral specimen, (ii) photograph of the bone plate, and (iii) bone ultrasound transducer [82]; (**c**) (i) experimental apparatus for mechanical electrical impedance measurements, (ii) the dimensions and materials of the monitoring structure, and (iii) materials used for the experiment [83]; (**d**) bones with DBPS and NBPS configurations [84]; (**e**) compliant layer adaptive composite stacks (i) 0.0  mm CLACS, and (ii) 0.8  mm CLACS [85].

In fracture treatment, there is usually a consequence of non-union in 5–10% of cases, which means that newly generated bone from the bone defect is not connected to the peripheral bone [79], thus requiring follow-up treatment, which lasts a long time and is expensive [80]. One commonly used treatment is systemic low-magnitude, LMHF vibration, which promotes bone connection by applying a mechanical load. However, this method produces unwanted systemic effects throughout the body and may cause side effects through hormonal changes. Faced with this situation, Bradley D. Nelson et al, designed a piezoelectric bone fixation plate that can be implanted at the non-connected part of the bone (Figure 7a), realized the mechanical vibration of the non-connected area, and evaluated the effectiveness of the treatment by identifying the longitudinal trend in bone stiffness [81]. Using piezoelectric materials as both sensors and brakes, it has been proven through 10,000 fatigue tests that the material can maintain reliability in long-term bone regeneration tests over 8 weeks [82].

Piezoelectric materials are used for bone repair because the electric field generated by piezoelectric materials in the ultrasound state promotes osteoblast migration and differentiation [86]. The frequency of ultrasound to promote bone regeneration is generally in the megahertz range [87]. Therefore, understanding the degree of piezoelectric properties of the bone itself in the megahertz segment plays an important role in bone repair. A transducer is an instrument that uses the piezoelectric effect to convert electrical energy into sound energy or the opposite [88,89]. For this reason, Taiki Makino et al. developed an ultrasonic transducer using bovine femoral material for the measurement of ultrasonic radiation and the reception of bone (Figure 7b) [82]. The cortical bone of the cattle femur was made into a round plate with a diameter of 10 mm and a thickness of about 1 mm, and XRD (X-ray diffraction) was used to measure the arrangement of hydroxyapatite crystals in the sample to determine the position of the bone axis. Experiments have shown that the ultrasonic radiation of a bone transmitter and the evoked potential of the receiver are relatively small—one one-thousandth of the level of the PVDF transducer under the same diameter. The use of transducers has confirmed the existence of small piezoelectric effects and inverse piezoelectric effects in bone in the megahertz segment, and quantitative analysis is useful in the clinical research on utilizing ultrasound in the megahertz segment to treat bone repair [90].

Piezoelectric devices can use mechanical electrical impedance technology to reveal changes in the elasticity of materials by detecting changes in resistance [91], which was previously commonly used for the safe detection of the material structure of aircraft wings or spacecraft [92] and can also be used for bone detection. Hector A. Tinoco et al. designed and evaluated a piezoelectric sensing device for biological applications on bones (Figure 7c) [83]. The experiment selected two materials—human teeth and an aluminum cone embedded in the substrate—to mimic the shape of the alveolar bone, and three different materials were used as the matrix. Measuring the velocity–frequency response curve, the elastic changes in the matrix were obtained through impedance analysis during the two frequency windows. At the same time, it was found that the coupling of different types of materials has different sensitivities; therefore, this application can identify the healing of bone injury through the electromechanical signal conversion of piezoelectric devices, and it is necessary to measure its sensitivity in advance when applied to different parts of bone tissue.

Directly bonded piezo sensors (DBPSs) refer to piezoelectric lead zirconate titanate (PZT) bonded directly to the patient’s injured limb. Using mechanical electrical impedance technology [93,94], the impedance change generated at the bone injury at the connection point can be detected by the surface-bonded PZT patch [7,95,96], The high-frequency excitations generated can quantify the extent of the injury or the extent of the healing. However, direct bonding can adversely affect the patient’s limb [97]. To solve this problem, Shashank Srivastava et al. proposed a new non-bonded piezo sensor (NBPS) configuration which connects a PZT patch to the middle of an aluminum strip (Figure 7d) [84]. To avoid the mechanical tightening of clamps through screws, which may cause discomfort to patients and damage the PZT patch, shape memory alloy (SMA) wires were chosen for clamping. NBPS with SMA clamping and traditional jubilee clamping were compared in healthy and osteoporosis subjects, i.e., two different states of bone replicas, and the results proved the effectiveness of the test. It also provided the quantification of parameters during bone degeneration [98].

The problem of slow bone healing and nonunion in smokers and diabetic people has always been a difficult problem to solve clinically [99,100,101]. Direct currents have been found to effectively promote bone healing [102,103,104]. The use of piezoelectric materials can effectively collect the energy generated by the human body in motion and convert it into direct current [105]; however, it is challenging to overcome the generation of sufficient power at low voltage and low frequency [106,107]. Piezoelectric stack materials have been used to increase power at low voltages before, but they have not been used as biological support materials. E.D. Krech et al. designed CLACS consisting of five piezo sheets with a layer of low modulus epoxy in the middle of each of the two piezoelectric sheets (Figure 7e) [85]. The volume is guaranteed to remain constant through the package; then, the power at different frequencies, voltages, and resistors is measured. The higher the thickness of the compliant layer, the heavier the mechanical load. Additionally, the higher the frequency, the better the output power of the material. This study showed that the device is suitable for the frequency and load of bone healing.

## 4. Conclusions and Outlook

This review details piezoelectric materials and piezoelectric devices, as well as their mechanisms of action in bone regeneration, and summarizes work in bone regeneration (Table 1).

With an aging population and a proliferation of patients with refractory bone defects due to trauma and tumors, the need for bone graft surgery and bone implants needs to be urgently addressed. Although autologous bone grafting is the gold standard for treating bone injuries, the limited amount of autologous bone available to fill large areas of bone defects has led to the creation of bone engineering. Superior performance piezoelectric materials have similar piezoelectric properties to natural bone tissue and can provide a good electrochemical microenvironment for defective tissue without the use of external power sources and electrodes. With the advancement of research, various piezoelectric materials are becoming more suitable for bone defect repair through improvement and modification, providing new directions for the translation of tissue engineering technology into clinical practice. Using the body’s self-generated motions, such as arm swings, extensions, or walking, as well as very small displacements within the body (e.g., breathing, heartbeat, blood flow, blinking, or muscle stretch), piezoelectric nanogenerators can power biomedical devices such as pacemakers and artificial retinas. Piezoelectric materials can mimic the bioelectrical signals of bone tissue, promote the ability of osteoblasts to adhere, proliferate, and differentiate, stimulate osteogenesis, and thus achieve bone repair; they also represent promising bone implants that provide new ideas for bone repair in complex bone diseases. However, there are still some problems to be solved for actual clinical applications, presented subsequently.

1. In addition to the excellent piezoelectric properties where the electrical signal generated by the piezoelectric material can reach the threshold value for treating bone tissue, the piezoelectric material needs to present good biocompatibility, degradability, and accurate simulation of the extracellular matrix microenvironmental conditions of bone tissue as an implant, which necessitates higher requirements for the development of new biomaterials.

2. Despite the significant positive effects of piezoelectric materials and devices on bone regeneration, the exact mechanism of action is still not well defined.

Miniaturization, good biocompatibility, easy degradation, and excellent output performance are further goals to be pursued for such materials and devices. The further optimization of piezoelectric composites for bone repair is another research goal because composites formed by combining piezoelectric materials with other bone implant materials can overcome the deficiencies of piezoelectric materials themselves while retaining the piezoelectricity of the materials. The ultimate goal of piezoelectric materials and devices for bone repair is to achieve clinical applications that improve health care and quality of life for patients with bone injuries.

## Figures and Tables

**Table 1:** Types, properties, and outcomes of piezoelectric material and devices.

Materials/Devices	Properties	Advantages	Disadvantages	Ref.
sm-PENG	Promote osteogenic differentiation	Memory of shape	Lack of validation of animal experiments.	[66]
ISPG	Host-coupled bio-nanogenerator (HCBG) configured with a self-powered regional electrical environment for bone regeneration	Achieved biomechanical energy scavenging and electrical stimulation therapy	In vivo degradation properties are unclear, and if surgical removal is required, it will cause secondary damage to the organism.	[67]
GaN/AlGaN	Enhance bio regeneration	Rapid and superior bone repair in vivo	The specific mechanism of action can be studied in more depth.	[68]
PHBV	Simulate the structure and piezoelectric coefficient of natural cartilage	Promote the regeneration of cartilage	The specific mechanism of action can be studied in more depth.	[70]
PLLA	Remote-controlled electrical stimulation	Repair mouse skull defects	The output performance of the device can continue to be optimized.	[71]
Piezoelectric fixation plate	Using piezoelectric materials as both sensors and brakes	Realize the mechanical vibration of non-connected areas and evaluate the effectiveness of the treatment	Inconvenient to carry.	[81]
An ultrasonic transducer using bovine femoral material	Measure the ultrasonic radiation and reception of bone	Measure the piezoelectric properties of the bone in the megahertz segment	In vivo degradation properties are unclear, and if surgical removal is required, it will cause secondary damage to the organism.	[82]
A piezoelectric sensing device for biological applications on bones.	Uses mechanical electrical impedance technology	Detect the healing of the bone injury	Lack of validation of animal experiments.	[83]
NBPS	Avoid the mechanical tightening of clamps through screws that may cause discomfort to patients and damage the PZT patch	Provides quantification of parameters during bone degeneration	The explanation of the mechanism needs to be improved.	[84]
CLACS	Generate direct current under the frequency and load of bone healing	Solve slow bone healing and nonunion	The explanation of the mechanism needs to be improved.	[85]

## Data Availability

Publicly available datasets were analyzed in this study. This data can be found here: https://www.webofscience.com/wos/alldb/citation-report/a2bd0068-059a-4d69-9fcb-91374739b09a-64581b12, accessed on 1 December 2022.

## References

[1] Mohammadkhah M., Marinkovic D., Zehn M., Checa S. (2019). A review on computer modeling of bone piezoelectricity and its application to bone adaptation and regeneration. Bone.

[2] Ribeiro C., Correia D.M., Rodrigues I., Guardão L., Guimarães S., Soares R., Lanceros-Méndez S. (2017). In vivo demonstration of the suitability of piezoelectric stimuli for bone reparation. Mater. Lett..

[3] Silva C.A., Fernandes M.M., Ribeiro C., Lanceros-Mendez S. (2022). Two- and three-dimensional piezoelectric scaffolds for bone tissue engineering. Colloids Surf. B Biointerfaces.

[4] Vasquez-Sancho F., Abdollahi A., Damjanovic D., Catalan G. (2018). Flexoelectricity in Bones. Adv. Mater..

[5] Samadi A., Salati M.A., Safari A., Jouyandeh M., Barani M., Singh Chauhan N.P., Golab E.G., Zarrintaj P., Kar S., Seidi F. (2022). Comparative review of piezoelectric biomaterials approach for bone tissue engineering. J. Biomater. Sci. Polym. Ed..

[6] Tandon B., Blaker J.J., Cartmell S.H. (2018). Piezoelectric materials as stimulatory biomedical materials and scaffolds for bone repair. Acta Biomater..

[7] Jacob J., More N., Kalia K., Kapusetti G. (2018). Piezoelectric smart biomaterials for bone and cartilage tissue engineering. Inflamm. Regen..

[8] D’Alessandro D., Ricci C., Milazzo M., Strangis G., Forli F., Buda G., Petrini M., Berrettini S., Uddin M.J., Danti S. (2021). Piezoelectric signals in vascularized bone regeneration. Biomolecules.

[9] Fortuna L., Buscarino A. (2022). Smart Materials. Materials.

[10] Curie P.C.J. (1880). Development by pressure of polar electricity in hemihedral crystals with inclined faces. Bull. Soc..

[11] Shamos M.H., Lavine L.S. (1967). Piezoelectricity as a fundamental property of biological tissues. Nature.

[12] Park J.B., Kelly B.J., Kenner G.H., von Recum A.F., Grether M.F., Coffeen W.W. (1981). Piezoelectric ceramic implants: In vivo results. J. Biomed. Mater. Res..

[13] Ali F., Raza W., Li X., Gul H., Kim K.-H. (2019). Piezoelectric energy harvesters for biomedical applications. Nano Energy.

[14] Wang L., Wang Z.L. (2021). Advances in piezotronic transistors and piezotronics. Nano Today.

[15] Peng B., Lu Q., Tang H., Zhang Y., Cheng Y., Qiu R., Guo Y., Zhou Z., Liu M. (2022). Large in-plane piezo-strain enhanced voltage control of magnetic anisotropy in Si-compatible multiferroic thin films. Mater. Horiz..

[16] Smith M., Kar-Narayan S. (2022). Piezoelectric polymers: Theory, challenges and opportunities. Int. Mater. Rev..

[17] Barros de Freitas R.L., Sakamoto W.K., Scarin Freitas L.P., Castro F., Lima Filho A.P., Kitano C., de Carvalho A.A. (2018). Characterization of PZT/PVDF composite film as functional material. IEEE Sens. J..

[18] Guo S.-L., Lai S.-N., Wu J.M. (2021). Strain-induced ferroelectric heterostructure catalysts of hydrogen production through piezophototronic and piezoelectrocatalytic system. ACS Nano.

[19] Xu Q., Gao X., Zhao S., Liu Y.-N., Zhang D., Zhou K., Khanbareh H., Chen W., Zhang Y., Bowen C. (2021). Construction of io-piezoelectric platforms: From structures and synthesis to applications. Adv. Mater..

[20] Zheng L., Huo X., Wang R., Wang J., Jiang W., Cao W. (2013). Large size lead-free (Na,K)(Nb,Ta)O-3 piezoelectric single crystal: Growth and full tensor properties. Crystengcomm.

[21] Maruska H.P., Tietjen J.J. (1969). Preparation and properties of vapor-deposited single-crystalline gan. Appl. Phys. Lett..

[22] Dingle R., Shaklee K.L., Leheny R.F., Zetterstrom R.B. (1971). Stimulated emission and laser action in gallium nitride. Appl. Phys. Lett..

[23] Qamar A., Dao D.V., Dinh T., Iacopi A., Walker G., Phan H.-P., Hold L., Dimitrijev S. (2017). Piezo-Hall effect and fundamental piezo-Hall coefficients of single crystal n-type 3C-SiC(100) with low carrier concentration. Appl. Phys. Lett..

[24] Bagnall D.M., Chen Y.F., Zhu Z., Yao T., Koyama S., Shen M.Y., Goto T. (1997). Optically pumped lasing of ZnO at room temperature. Appl. Phys. Lett..

[25] Chu S., Wang G., Zhou W., Lin Y., Chernyak L., Zhao J., Kong J., Li L., Ren J., Liu J. (2011). Electrically pumped waveguide lasing from ZnO nanowires. Nat. Nanotechnol..

[26] Chu S.Y., Chen T.Y. (2004). Fabrication of modified lead titanate piezoceramics with zero temperature coefficient and its application on SAW devices. IEEE Trans. Ultrason. Ferroelectr. Freq. Control.

[27] Cao W.P., Sheng J., Qiao Y.L., Jing L., Liu Z., Wang J., Li W.L. (2019). Optimized strain with small hysteresis and high energy-storage density in Mn-doped NBT-ST system. J. Eur. Ceram. Soc..

[28] Wu H.-S., Murti B.T., Singh J., Yang P.-K., Tsai M.-L. (2022). Prospects of metal-free perovskites for piezoelectric applications. Adv. Sci..

[29] Chorsi M.T., Curry E.J., Chorsi H.T., Das R., Baroody J., Purohit P.K., Ilies H., Nguyen T.D. (2019). Piezoelectric biomaterials for sensors and actuators. Adv. Mater..

[30] Chang C., Tran V.H., Wang J., Fuh Y.K., Lin L. (2010). Direct-write piezoelectric polymeric nanogenerator with high energy conversion efficiency. Nano Lett.

[31] Datta A., Choi Y.S., Chalmers E., Ou C., Kar-Narayan S. (2017). Piezoelectric nylon-11 nanowire arrays grown by template wetting for vibrational energy harvesting applications. Adv. Funct. Mater..

[32] Pi Z., Zhang J., Wen C., Zhang Z.-B., Wu D. (2014). Flexible piezoelectric nanogenerator made of poly(vinylidenefluoride-co-trifluoroethylene) (PVDF-TrFE) thin film. Nano Energy.

[33] Qian J., Peng R., Shen Z., Jiang J., Xue F., Yang T., Chen L., Shen Y. (2019). Interfacial coupling boosts giant electrocaloric effects in relaxor polymer nanocomposites: In situ characterization and phase-field simulation. Adv. Mater..

[34] Tai Y., Yang S., Yu S., Banerjee A., Myung N.V., Nam J. (2021). Modulation of piezoelectric properties in electrospun PLLA nanofibers for application-specific self-powered stem cell culture platforms. Nano Energy.

[35] Qu X., Ma X., Shi B., Li H., Zheng L., Wang C., Liu Z., Fan Y., Chen X., Li Z. (2021). Refreshable braille display system based on triboelectric nanogenerator and dielectric elastomer. Adv. Funct. Mater..

[36] Liu K., Cao Y., Wang G., Zhang W., Chen W., Gao X. (2018). A novel photoacoustic spectroscopy gas sensor using a low cost polyvinylidene fluoride film. Sens. Actuators B-Chem..

[37] Wang A., Hu M., Zhou L., Qiang X. (2018). Self-powered wearable pressure sensors with enhanced piezoelectric properties of aligned p(VDF-TrFE)/MWCNT composites for monitoring human physiological and muscle motion signs. Nanomaterials.

[38] Martin A.J.P. (1941). Tribo-electricity in wool and hair. Proc. Phys. Soc..

[39] Fukada E., Yasuda I. (1957). On the piezoelectric effect of bone. J. Phys. Soc. Jpn..

[40] Fukada E., Ueda H., Rinaldi R. (1976). Piezoelectric and related properties of hydrated collag. Biophys. J..

[41] Fukada E., Hara K. (1969). Piezoelectric effect in blood vessel walls. J. Phys. Soc. Jpn..

[42] Derossi D., Pastacaldi P., Domenici C. (1986). Piezoelectric properties of dry human-skin. IEEE Trans. Electr. Insul..

[43] Fukada E., Ueda H. (1970). Piezoelectric effect in muscle. Jpn. J. Appl. Phys..

[44] Athenstaedt H. (1970). Permanent longitudinal electric polarization and pyroelectric behaviour of collagenous structures and nervous tissue in man and other vertebrates. Nature.

[45] Fukada E. (1996). Piezoelectricity of biopolymers. Biorheology.

[46] Chow W.S., Ishak Z.A.M. (2020). Smart polymer nanocomposites: A review. Express Polym. Lett..

[47] Li J., Li Y., Zhu D., Wang Q., Zhang Y., Zhu Y., Li M. (2016). Magnetoelectric effect modulation in a PVDF/Metglas/PZT composite by applying DC electric fields on the PZT phase. J. Alloys Compd..

[48] Wang J., Houwman E., Salm C., Minh N., Vergeer K., Schmitz J. (2017). Process induced poling and plasma induced damage of thin film PZT. Microelectron. Eng..

[49] Rasoolzadeh M., Sherafat Z., Vahedi M., Bagherzadeh E. (2022). Structure dependent piezoelectricity in electrospun PVDF-SiC nanoenergy harvesters. J. Alloys Compd..

[50] Zhang Y., Liu Y., Wang Z.L. (2011). Fundamental theory of piezotronics. Adv. Mater..

[51] Wu W., Wen X., Wang Z.L. (2013). Taxel-addressable matrix of vertical-nanowire piezotronic transistors for active and adaptive tactile imaging. Science.

[52] Wen X., Wu W., Ding Y., Wang Z.L. (2013). Piezotronic effect in flexible thin-film based devices. Adv. Mater..

[53] Liu H., Hua Q., Yu R., Yang Y., Zhang T., Zhang Y., Pan C. (2016). A bamboo-like gan microwire-based piezotronic memristor. Adv. Funct. Mater..

[54] Wang C.-H., Liao W.-S., Ku N.-J., Li Y.-C., Chen Y.-C., Tu L.-W., Liu C.-P. (2014). Effects of free carriers on piezoelectric nanogenerators and piezotronic devices made of gan nanowire arrays. Small.

[55] Yu R., Dong L., Pan C., Niu S., Liu H., Liu W., Chua S., Chi D., Wang Z.L. (2012). Piezotronic effect on the transport properties of GaN nanobelts for active flexible electronics. Adv. Mater..

[56] Zhang J., Meguid S.A. (2015). On the piezoelectric potential of gallium nitride nanotubes. Nano Energy.

[57] Yu R., Wang X., Wu W., Pan C., Bando Y., Fukata N., Hu Y., Peng W., Ding Y., Wang Z.L. (2015). Temperature dependence of the piezophototronic effect in CdS nanowires. Adv. Funct. Mater..

[58] Zhou Y.S., Wang K., Han W., Rai S.C., Zhang Y., Ding Y., Pan C., Zhang F., Zhou W., Wang Z.L. (2012). Vertically aligned CdSe nanowire arrays for energy harvesting and piezotronic devices. ACS Nano.

[59] Peng Y., Que M., Lee H.E., Bao R., Wang X., Lu J., Yuan Z., Li X., Tao J., Sun J. (2019). Achieving high-resolution pressure mapping via flexible GaN/ZnO nanowire LEDs array by piezo-phototronic effect. Nano Energy.

[60] Li X., Wei X., Xu T., Pan D., Zhao J., Chen Q. (2015). Remarkable and crystal-structure-dependent piezoelectric and piezoresistive effects of InAs nanowires. Adv. Mater..

[61] Ku N.-J., Huang J.-H., Wang C.-H., Fang H.-C., Liu C.-P. (2012). Crystal face-dependent nanopiezotronics of an obliquely aligned inn nanorod array. Nano Lett..

[62] Wu J.M., Chen K.-H., Zhang Y., Wang Z.L. (2013). A self-powered piezotronic strain sensor based on single ZnSnO_3_ microbelts. RSC Adv..

[63] Wu J.M., Chen C.-Y., Zhang Y., Chen K.-H., Yang Y., Hu Y., He J.-H., Wang Z.L. (2012). Ultrahigh sensitive piezotronic strain sensors based on a ZnSnO_3_ nanowire/microwire. ACS Nano.

[64] Hou T.-C., Yang Y., Lin Z.-H., Ding Y., Park C., Pradel K.C., Chen L.-J., Wang Z.L. (2013). Nanogenerator based on zinc blende CdTe micro/nanowires. Nano Energy.

[65] Wang Z.L., Song J.H. (2006). Piezoelectric nanogenerators based on zinc oxide nanowire arrays. Science.

[66] Zhang Y., Lingling X., Liu Z., Cui X., Xiang Z., Bai J., Jiang D., Xue J., Wang C., Lin Y. (2021). Self-powered pulsed direct current stimulation system for enhancing osteogenesis in MC3T3-E1. Nano Energy.

[67] Yu B., Qiao Z., Cui J., Lian M., Han Y., Zhang X., Wang W., Yu X., Yu H., Wang X. (2021). A host-coupling bio-nanogenerator for electrically stimulated osteogenesis. Biomaterials.

[68] Zhang C., Wang W., Hao X., Peng Y., Zheng Y., Liu J., Kang Y., Zhao F., Luo Z., Guo J. (2021). A novel approach to enhance bone regeneration by controlling the polarity of GaN/AlGaN heterostructures. Adv. Funct. Mater..

[69] Qiao Z., Lian M., Liu X., Zhang X., Han Y., Ni B., Xu R., Yu B., Xu Q., Dai K. (2022). Electreted sandwich membranes with persistent electrical stimulation for enhanced bone regeneration. ACS Appl. Mater. Interfaces.

[70] Jacob J., More N., Mounika C., Gondaliya P., Kalia K., Kapusetti G. (2019). Smart piezoelectric nanohybrid of poly(3-hydroxybutyrate-co-3-hydroxyvalerate) and barium titanate for stimulated cartilage regeneration. ACS Appl. Bio Mater..

[71] Das R., Curry E.J., Le T.T., Awale G., Liu Y., Li S., Contreras J., Bednarz C., Millender J., Xin X. (2020). Biodegradable nanofiber bone-tissue scaffold as remotely-controlled and self-powering electrical stimulator. Nano Energy.

[72] Yu P., Ning C., Zhang Y., Tan G., Lin Z., Liu S., Wang X., Yang H., Li K., Yi X. (2017). Bone-inspired spatially specific piezoelectricity induces bone regeneration. Theranostics.

[73] Zhao F., Zhang C., Liu J., Liu L., Cao X., Chen X., Lei B., Shao L. (2020). Periosteum structure/function-mimicking bioactive scaffolds with piezoelectric/chem/nano signals for critical-sized bone regeneration. Chem. Eng. J..

[74] Ahmadi N., Kharaziha M., Labbaf S. (2020). Core–shell fibrous membranes of PVDF–Ba_0.9_Ca_0.1_TiO_3_/PVA with osteogenic and piezoelectric properties for bone regeneration. Biomed. Mater..

[75] Pai S., Kwon J., Liang B., Cho H., Soghrati S. (2021). Finite element analysis of the impact of bone nanostructure on its piezoelectric response. Biomech. Model. Mechanobiol..

[76] Marino A.A., Gross B.D. (1989). Piezoelectricity in cementum, dentine and bone. Arch. Oral Biol..

[77] Akbari N., Khorshidi S., Karkhaneh A. (2022). Effect of piezoelectricity of nanocomposite electrospun scaffold on cell behavior in bone tissue engineering. Iran. Polym. J..

[78] More N., Kapusetti G. (2017). Piezoelectric material—A promising approach for bone and cartilage regeneration. Med. Hypotheses.

[79] Polley C., Distler T., Detsch R., Lund H., Springer A., Boccaccini A.R., Seitz H. (2020). 3D Printing of piezoelectric barium titanate-hydroxyapatite scaffolds with interconnected porosity for bone tissue engineering. Materials.

[80] Liu W., Yang D., Wei X., Guo S., Wang N., Tang Z., Lu Y., Shen S., Shi L., Li X. (2020). Fabrication of piezoelectric porous BaTiO3 scaffold to repair large segmental bone defect in sheep. J. Biomater. Appl..

[81] Nelson B.D., Karipott S.S., Guldberg R.E., Ong K.G. (2020). A piezoelectric bone fixation plate for in vivo application and monitoring of mechanical loading during fracture healing. Meas. Sci. Technol..

[82] Makino T., Nakamura T., Bustamante L., Takayanagi S., Koyama D., Matsukawa M. (2020). Piezoelectric and inversely piezoelectric responses of bone tissue plates in the megahertz range. IEEE Trans. Ultrason. Ferroelectr. Freq. Control.

[83] Tinoco H.A., Cardona C.I., Peña F.M., Gomez J.P., Roldan-Restrepo S.I., Velasco-Mejia M.A., Barco D.R. (2019). Evaluation of a piezo-actuated sensor for monitoring elastic variations of its support with impedance-based measurements. Sensors.

[84] Srivastava S., Bhalla S., Madan A. (2019). Shape memory alloy actuation of non-bonded piezo sensor configuration for bone diagnosis and impedance based analysis. Biomed. Eng. Lett..

[85] Krech E.D., Cadel E.S., Barrett R.M., Friis E.A. (2018). Effect of compliant layers within piezoelectric composites on power generation providing electrical stimulation in low frequency applications. J. Mech. Behav. Biomed. Mater..

[86] Hosokawa A. (2020). Change of piezoelectric signal in cancellous bone with ultrasound irradiation angle. Jpn. J. Appl. Phys..

[87] Li Y., Liao C., Tjong S.C. (2019). Electrospun polyvinylidene fluoride-based fibrous scaffolds with piezoelectric characteristics for bone and neural tissue engineering. Nanomaterials.

[88] Lakes R. (1980). The role of gradient effects in the piezoelectricity of bone. IEEE Trans. Biomed. Eng..

[89] Hosokawa A., Matsukawa M., Laugier P., Grimal Q. (2022). Piezoelectric and opto-acoustic material properties of bone. Bone Quantitative Ultrasound: New Horizons.

[90] KÖNig C.W., TrÜBenbach J., BÖHm P., Fritz J.A.N., Duda S.H., Pereira P.L. (2003). Magnetic resonance-guided transcortical biopsy of bone marrow lesions using a magnetic resonance imaging-compatible piezoelectric power drill. Investig. Radiol..

[91] Shuai C., Liu G., Yang Y., Yang W., He C., Wang G., Liu Z., Qi F., Peng S. (2020). Functionalized BaTiO_3_ enhances piezoelectric effect towards cell response of bone scaffold. Colloids Surf. B Biointerfaces.

[92] Shokrollahi H., Salimi F., Doostmohammadi A. (2017). The fabrication and characterization of barium titanate/akermanite nano-bio-ceramic with a suitable piezoelectric coefficient for bone defect recovery. J. Mech. Behav. Biomed. Mater..

[93] Gorodzha S.N., Muslimov A.R., Syromotina D.S., Timin A.S., Tcvetkov N.Y., Lepik K.V., Petrova A.V., Surmeneva M.A., Gorin D.A., Sukhorukov G.B. (2017). A comparison study between electrospun polycaprolactone and piezoelectric poly(3-hydroxybutyrate-co-3-hydroxyvalerate) scaffolds for bone tissue engineering. Colloids Surf. B Biointerfaces.

[94] Manohar C.S., Kumar B.S., Sadhu S.P.P., Srimadh S.K., Muthukumar V.S., Venketesh S., Varma K.B.R. (2019). Novel lead-free biocompatible piezoelectric hydroxyapatite (HA)—BCZT (Ba_0.85_Ca_0.15_Zr_0.1_Ti_0.9_O_3_) nanocrystal composites for bone regeneration. Nanotechnol. Rev..

[95] Pekovits K., Wildburger A., Payer M., Hutter H., Jakse N., Dohr G. (2012). Evaluation of Graft Cell Viability—Efficacy of Piezoelectric Versus Manual Bone Scraper Technique. J. Oral Maxillofac. Surg..

[96] Stroe M.C., Crolet J.M., Racila M. (2013). Mechanotransduction in cortical bone and the role of piezoelectricity: A numerical approach. Comput. Methods Biomech. Biomed. Eng..

[97] Balasandaram I., Bridle C., Holmes S. (2015). Piezoelectric surgery—Primary bone grafting in craniofacial trauma revisited. Int. J. Oral Maxillofac. Surg..

[98] Gellner V., Koele W., Wolf A., Gerstenberger C., Mokry M., Stammberger H., Tomazic P.V. (2017). A piezoelectric device for bone work in endoscopic anterior skull base surgery—A feasibility study in 15 patients. Clin. Otolaryngol..

[99] Samadi A., Hasanzadeh R., Azdast T., Abdollahi H., Zarrintaj P., Saeb M.R. (2020). Piezoelectric performance of microcellular polypropylene foams fabricated using foam injection molding as a potential scaffold for bone tissue engineering. J. Macromol. Sci. Part B.

[100] Park I.-Y., Shimizu Y., O’Connor K.N., Puria S., Cho J.-H. (2011). Comparisons of electromagnetic and piezoelectric floating-mass transducers in human cadaveric temporal bones. Hear. Res..

[101] Zheng T., Zhao H., Huang Y., Gao C., Zhang X., Cai Q., Yang X. (2021). Piezoelectric calcium/manganese-doped barium titanate nanofibers with improved osteogenic activity. Ceram. Int..

[102] Reis J., Frias C., Capela-Silva F., Potes J., Botelho L., Castro C., Marques A., Simões J. (2012). Piezoelectric actuators for bone mechanical stimulation: Exploring the concept. J. Biomech..

[103] Aschero G., Gizdulich P., Mango F., Romano S.M. (1996). Converse piezoelectric effect detected in fresh cow femur bone. J. Biomech..

[104] Mouraret S., Houschyar K.S., Hunter D.J., Smith A.A., Jew O.S., Girod S., Helms J.A. (2014). Cell viability after osteotomy and bone harvesting: Comparison of piezoelectric surgery and conventional bur. Int. J. Oral Maxillofac. Surg..

[105] Augello M., Deibel W., Nuss K., Cattin P., Jürgens P. (2018). Comparative microstructural analysis of bone osteotomies after cutting by computer-assisted robot-guided laser osteotome and piezoelectric osteotome: An in vivo animal study. Lasers Med. Sci..

[106] Tanaka S.M. (1999). A new mechanical stimulator for cultured bone cells using piezoelectric actuator. J. Biomech..

[107] Younes R., Nasseh I., Lahoud P., Wassef E., Dagher M. (2017). Bone lid technique using a piezoelectric device for the treatment of a mandibular bony lesion. Case Rep. Dent..

